# Exercise training with negative pressure ventilation improves exercise capacity in patients with severe restrictive lung disease: a prospective controlled study

**DOI:** 10.1186/1465-9921-14-22

**Published:** 2013-02-19

**Authors:** Shu-Chuan Ho, Horng-Chyuan Lin, Han-Pin Kuo, Li-Fei Chen, Te-Fang Sheng, Wen-Ching Jao, Chun-Hua Wang, Kang-Yun Lee

**Affiliations:** 1 Department of Thoracic Medicine, Chang Gung Memorial Hospital, Chang Gung University College of Medicine, 199 Tun-Hwa North Road, Taipei, Taiwan; 2 Chang Gung University, Department of Medicine, College of Medicine, Taoyuan, Taiwan; 3 School of Respiratory Therapy, College of Medicine, Taipei Medical University, Taipei, Taiwan

**Keywords:** Restrictive lung disease, Negative pressure ventilation, Exercise training, Exercise capacity, Health-related quality of life

## Abstract

**Background:**

Exercise training is of benefit for patients with restrictive lung disease. However, it tends to be intolerable for those with severe disease. We examined whether providing ventilatory assistance by using negative pressure ventilators (NPV) during exercise training is feasible for such patients and the effects of training.

**Methods:**

36 patients with restrictive lung disease were prospectively enrolled for a 12-week multidisciplinary rehabilitation program. During this program, half of them (n:18; 60.3 ± 11.6 years; 6 men; FVC: 32.5 ± 11.7% predicted ) received regular sessions of exercise training under NPV, whilst the 18 others (59.6 ± 12.3 years; 8 men; FVC: 37.7 ± 10.2% predicted) did not. Exercise capacity, pulmonary function, dyspnea and quality of life were measured. The primary endpoint was the between-group difference in change of 6 minute-walk distance (6MWD) after 12 weeks of rehabilitation.

**Results:**

All patients in the NPV-exercise group were able to tolerate and completed the program. The between-group differences were significantly better in the NPV-exercise group in changes of 6MWD (34.1 ± 12.7 m vs. -32.5 ± 17.5 m; *P* = 0.011) and St George Score (−14.5 ± 3.6 vs. 11.8 ± 6.0; P < 0.01). There was an improvement in dyspnea sensation (Borg’s scale, from 1.4 ± 1.5 point to 0.8 ± 1.3 point, *P* = 0.049) and a small increase in FVC (from 0.85 ± 0.09 L to 0.91 ± 0.08 L, *P* = 0.029) in the NPV-exercise group compared to the control group.

**Conclusion:**

Exercise training with NPV support is feasible for patients with severe restrictive lung diseases, and improves exercise capacity and health-related quality of life.

## Background

Patients with restrictive lung diseases (RLD) often suffer from dyspnea, fatigue and impairments in muscle force, exercise tolerance, and activity of daily life (ADL), leading to a decrease in health-related quality of life (QoL) [1-3]. This is due to a complicated combination of impairments in ventilation and gas exchange and a number of secondary changes such as peripheral muscle, cardiac, nutritional, and psychological impairments [4]. Following the consensus of pulmonary rehabilitation (PR) in chronic obstructive pulmonary disease (COPD) [5,6], evidence is growing that patients with RLD, which share many manifestations and physiological impairments with COPD, may also benefit from PR. Early randomized control studies have reported an improvement in exercise capacity and QoL after 8–10 weeks of exercise training [7,8]. The benefits disappeared after 6 months, however this could be overcome by longer programs extending to 24 weeks which showed even better results [9]. Although the latter studies were weakened by the non-randomized and non-controlled design, the message is important. As many of those likely to be enrolled tend to have early and profound exercise-induced oxygen desaturation, dyspnea and peripheral muscle weakness, an extended program may offset the low initial training load [4]. Alternatively, exercise training with some assistance, and in particular ventilatory support, may be useful, although this has not yet been extensively tested.

Non-invasive mechanical ventilatory techniques include the use of negative and positive pressure ventilators. Using non-invasive positive pressure ventilators (NPPV) as an aid during exercise training has been reported to enable a higher intensity of training for patients with severe COPD, leading to larger improvements in exercise performance [10,11]. In patients with severe RLD, NPPV during exercise has been shown to improve oxygenation and exercise tolerance [12], whilst the effect on exercise training has not been studied. Nevertheless, some practical drawbacks such as discomfort from the mouthpiece or mask [10] can result in a substantial number of patients dropping out [13]. Alternatively, negative pressure ventilators (NPV) (iron lungs) have been shown to be as effective as NPPV in certain conditions such as acute exacerbation of COPD, and to play a role in those who cannot tolerate a facial mask [14]. Short-term intermittent NPV has been demonstrated to improve ventilation and increase PaO_2_ in patients with severe hypercapnic COPD [15].

We hypothesized that an exercise training PR program with NPV support in patients with severe RLD would increase exercise capacity, decrease dyspnea sensation, and improve the QoL. We tested this hypothesis by conducting a prospective controlled study with exercise capacity as the primary endpoint.

## Materials and methods

### Subjects

This prospective, non-randomized, controlled study was carried out from 30 November 2008 to 30 May 2010 at Chang Gung Memorial Hospital, a university hospital in Linko, Taiwan. All patients with severe RLD referred by the chest physicians from the outpatient department for pulmonary rehabilitation were eligible for inclusion if they were clinically stable and presented with forced vital capacity (FVC) ≤ 50% predicted. Patients were excluded if they had a coexisting airway obstruction (forced expiratory volume in one second (FEV_1_)/FVC ≤ 70%), other respiratory diseases such as asthma, tuberculosis, COPD, bronchiectasis, cancer, and acute infection in the last 3 months. The decision to receive NPV-supported exercise training was made by the chest physician after discussing the program with the patients and their family. The protocol was approved by the institutional review board (CGMH 97-1032B). Each patient gave informed consent to participate.

### Study design

All patients attended a 12-week, twice-a-week, outpatient, multidiscipline PR program, consisting of breathing retraining, limb exercise training and an education program (lectures and discussions on RLD, medication use and nutrition, relaxation techniques, and home exercise). The patients in the NPV-exercise group also received endurance exercise training consisting of 20 minutes of submaximal cycle ergometry, combined strength/resistance training of the upper limbs through a series of 10 repetitions against progressive resistance, and specific training of respiratory muscles and breathing technique exercises of the thoracic and abdominal muscles, guided by Borg’s scale (score between 4 to 6 for dyspnea and fatigue), with the support of NPV (control + sign model delivered by −20 cmH_2_O delivery pressure and +3 cmH_2_O base pressure, 12 cycles per minute, inspiratory time to expiratory time (I/E) ratio 2.5). To determine the exercise capacity, a symptom-limited bicycle ergometry test as previous reported [16] was performed using a ramp 10, 15, or 20 protocol, depending on the patient’s fitness. The load was increased every minute by 10, 15, or 20 Watts, respectively, in such a way that patients could reach their maximal workload within 10 minutes. The test was terminated on the basis of the patient’s symptoms or at the physician’s discretion. Maximal workload in Watts at maximal performance was taken for analysis. 40–60% of the maximal workload was used as the intensity of training. All patients were established on an unsupervised home endurance exercise program of 20–30 minutes walk, continuously or intermittently (in sessions lasting 10 or more minutes) per day, guided by 40–60% of target heart rate or 4–6 on the modified Borg scale, with the aim of achieving 3 to 5 exercise sessions per week. Oxygen was provided by a nasal cannula to maintain SatO_2_ ≥ 90% during the whole PR course for all patients.

### Clinical assessments

FEV_1_, FVC and FEV_1_/FVC were recorded using a spirometer (ST-250, Fukuda Sangyo Co. Ltd.) and 6-minute walk test (6MWT) was performed with a pulse oximeter by a finger transducer (Criticare Systems Inc.) according to the ATS guidelines [17,18]. The dyspnea score was rated by patients according to a modified Borg's scale [19], in which words describing increasing degrees of dyspnea were assigned to numbers between 0 (no dyspnea) and 10 ( maximal dyspnea).

Health-related quality of life (HRQoL) was assessed using St. George’s Respiratory Questionnaire (SGRQ), a self-administered questionnaire measuring impaired health and perceived HRQoL for airways disease. It consists of 76 items including symptoms, activity, impact and total scores. The scores range from 0 (no impairment) to 100 (the worst impairment), in which higher scores connoted greater distress and worse HRQoL [20].

Maximal voluntary isometric muscle force of the right and left extremity of the following muscle groups was measured: extension of the knees and flexion of the elbows, using a handheld dynamometer (Hoggan Health Industry, West Jordon, Utah USA). The test was repeated three times, and the best result of either side was used in the analyses.

### Statistic analysis

Descriptive statistics were expressed as mean ± SD. Between group changes in pulmonary functions, 6MWD, Borg score and SGRQ scores were analyzed using the Mann–Whitney U test. 6MWD, FVC, FEV_1_ and SGRQ at enrollment, and after 4 weeks, 12 weeks and 52 weeks were compared using the Friedman test with Dunn’s multiple comparison post-test for the NPV-exercise group. SGRQ before and after the 12-week PR program was compared by the Wilcoxon signed rank test. Data were presented as mean ± SD. A *p* value less than 0.05 was considered significant. Data analyses were performed using the GraphPad Prism 5.01 software package (GraphPad Software, San Diego, CA).

## Results

### Study population

Eighteen patients (6 males and 12 females, mean age 60.3 ± 11.6 years; FVC: 32.5 ± 11.7% predicted) in the NPV-exercise group, and 18 patients (8 males and 10 females, mean age 59.6 ± 12.3 years; FVC: 37.7 ± 10.2% predicted) in the control group were enrolled. Before entry into the program, most of the patients could not tolerate exercise training without NPV support. All of the eighteen patients in the NPV-exercise group completed the 12-week NPV-exercise program.

The baseline characteristics of both groups are shown in Table 1. There were no significant differences in age, gender, BMI, FVC, FEV_1_, FEV_1_/FVC, 6MWD and Borg scales between the two groups. Eight patients had chest wall deformities and 10 patients had interstitial lung diseases in the NPV-exercise group, while 6 patients had chest wall deformities and 12 patients had interstitial lung disease in the control group. Patients in both groups had low BMI (19.7 ± 5.0 kg/m^2^ vs. 21.5 ± 4.6 kg/m^2^), poor SGRQ scores (42.6 ± 4.6 vs. 44.6 ± 4.8) and poor exercise tolerance (6MWD, 362.8 ± 99.4 m vs. 371.3 ± 77.9 m) with equal severity. After the end of the 12-week study, observational data were collected for those who regularly came back to our OPD until 52 weeks. These patients (*n* = 14) were all in the NPV-exercise. The ratio between IPF and CWD was almost unchanged at each time of evaluation, being 10/8, 8/5, 10/8 and 7/7 at week 0, 4, 12 and 52. As most of the patients in the control group were lost to follow-up by 52 weeks, their clinical data were not available for analysis.

**Table 1:** Characteristics of patients with restrictive lung disease in both groups

**Characteristics**	**Non-NPV exercise group**	**NPV-exercise group**
Number of patients	18	18
Age, years	59.6 ± 12.3	60.3 ± 11.6
Gender, M/F	8/10	6/12
BMI, kg/m^2^	19.7 ± 5.0	21.5 ± 4.6
FVC, L	1.05 ± 0.34	0.85 ± 0.37
FVC, % predicted	37.7 ± 10.2	32.5 ± 11.7
FEV_1_, L	0.88 ± 0.26	0.72 ± 0.32
FEV_1_, % predicted	42.2 ± 2.4	36.9 ± 13.6
FEV_1_/FVC, %	84.2 ± 8.3	83.1 ± 13.6
6MWD, m	362.8 ± 99.4	371.3 ± 77.9
Borg score, median(range)	0.5 (0–4)	1.25 (0–4)

BMI, Body mass index; 6MWD, 6-min walk distance.

### Changes in 6MWD and dyspnea sensation

**Figure 1:**
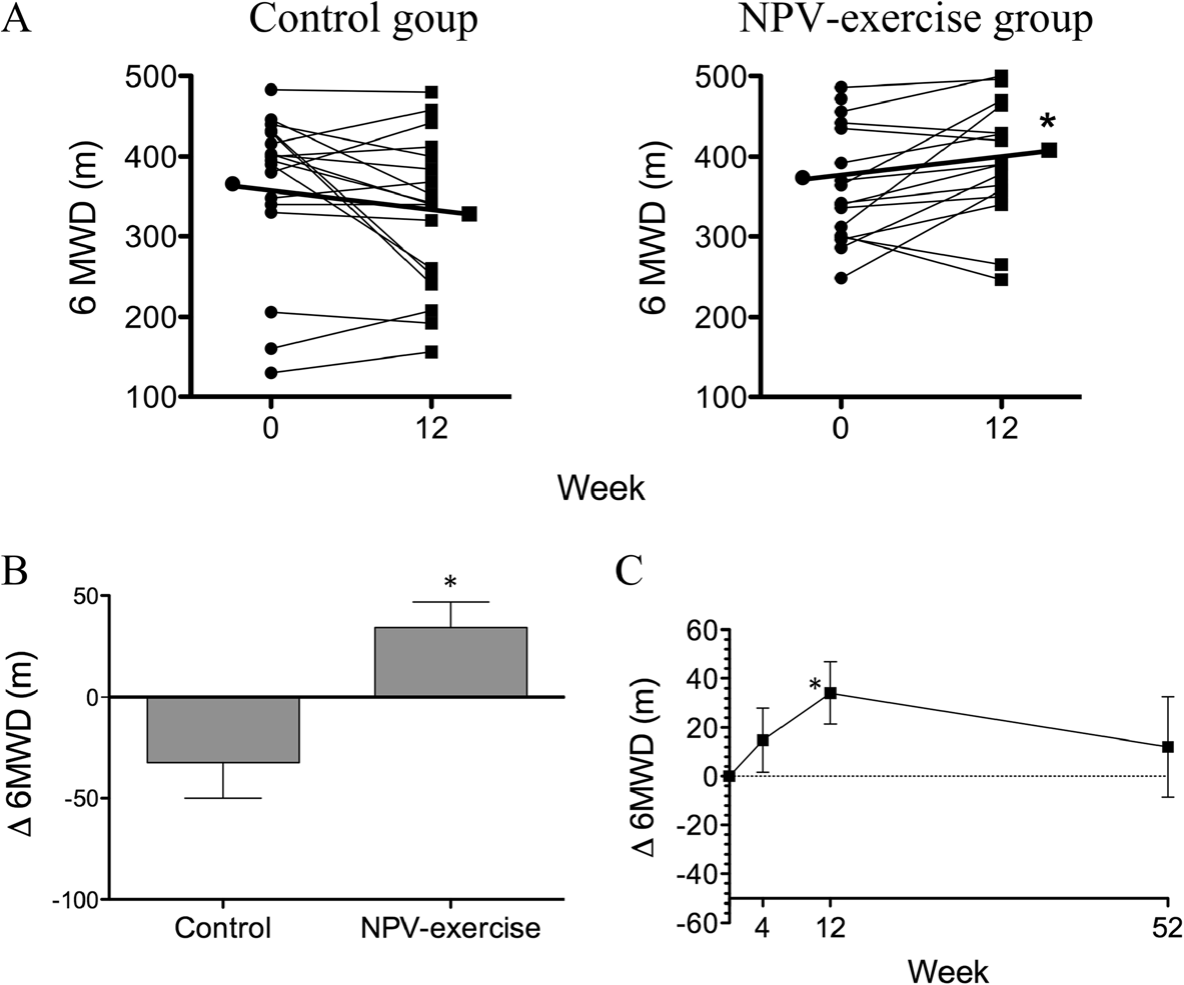
**Effects of NPV-exercise training vs. control on 6-minute walk distance. A.** Individual changes of 6MWD in both groups before and after the 12-week course (Control, *n* = 18; NPV-exercise, *n* = 18). **B.** Comparisons of delta-6MWD (Δ6MWD; changes from the base line after 12 weeks; mean ± SD) between both groups (Control, *n* = 18; NPV-exercise, *n* = 18). **C.** Time course of 6MWD in the NPV-exercise group (*n* = 14) during the 12 months.

Table 2 shows the effect of the PR program on 6MWT, pulmonary function and quality of life. At the end of 12 weeks, patients in the NPV-exercise group had an increase in 6MWD by 34.1 ± 12.7 m (Table 2 and Figure 1A, 371.3 ± 77.9 m vs. 405.4 ± 78.1 m; *P* = 0.016). In contrast, patients in the control group had a trend of a decrease by 32.5 ± 17.5 m (362.8 ± 99.4 m vs. 330.3 ± 94.4 m; *P* = 0.080). The between group difference was significant (Figure 1B, *P* = 0.011). The increase in 6MWD in the NPV-exercise group started at 4 weeks, reached statistical significance at 12 weeks, and disappeared at 52 weeks (Figure 1C).

**Table 2:** Changes in pulmonary function, 6-minute walk test, and health-related quality of life before and after 12 weeks of pulmonary rehabilitation

	**Non-NPV-exercise group**		**NPV-exercise group**	
	**Baseline**	**12 weeks**	**Baseline**	**12 weeks**
	**(*n*** **= 18)**	**(*n*** **= 18)**	**(*n*** **= 18)**	**(*n*** **= 18)**
FVC, % predicted	37.7 ± 2.4	37.9 ± 3.2	32.6 ± 2.8	35.1 ± 2.7^a^
FEV_1_, % predicted	42.2 ± 2.9	43.9 ± 4.3	36.9 ± 3.2	40.3 ± 3.3^a^
FEV_1_/FVC, %	84.1 ± 8.6	83.6 ± 7.8	82.9 ± 9.4	83.5 ± 10.8
6MWD, m	362.8 ± 99.4	330.3 ± 94.4	371.3 ± 77.9	405.4 ± 78.1^a^
SaO2, % pre/post 6MWT	94.0/ 81.4	93.2/ 76.8	93.9/ 77.0	94.6/ 79.1
HR, pre/ post 6MWT	98.2/ 125.8	101.4/ 127.7	103.3/ 126.7	92.0/ 123.4
Borg, pre/ post 6MWT	0.5/ 5.0	1.5/ 7.0	1.25/ 5.0	0/ 4.5^a^
(range)	(0–4)/ (3–10)	(0–4)/ (2–9)	(0–4)/ (4–8)	(0–4)/ (1–8)
Limb muscle power				
R-upper limb, lb	32.9 ± 1.8^b^	32.3 ± 1.6^b^	31.0 ± 2.1^c^	33.5 ± 2.2^a, c^
L-upper limb, lb	30.2 ± 3.5^b^	33.0 ± 4.7^b^	29.6 ± 1.6^c^	30.3 ± 1.6^c^
R-low limb, lb	44.3 ± 3.9^b^	46.2 ± 4.9^b^	38.5 ± 3.0^c^	41.4 ± 2.3^c^
L-low limb. lb	41.2 ± 3.4^b^	41.3 ± 4.6^b^	38.2 ± 2.7^c^	40.7 ± 2.2^c^
SGRQ				
Symptom score	48.2 ± 6.0^e^	46.5 ± 6.1^e^	54.6 ± 5.0	24.5 ± 4.0^a^
Active score	61.6 ± 6.0^e^	72.3 ± 6.1^e^	53.7 ± 5.8	45.9 ± 5.6^a^
Impacts score	30.0 ± 4.2^e^	46.7 ± 7.0^a,e^	36.2 ± 5.7	22.6 ± 5.1^a^
Total score	42.6 ± 4.6^e^	54.4 ± 4.9^e^	44.6 ± 4.8	30.0 ± 4.4^a^

Values are mean ± SD.

^a^ p < 0.05, baseline vs. 12 weeks.

^b^ only 6 patients in the non-NPV exercise group received limb muscle tests.

^c^ 17 patients in the NPV exercise group received limb muscle tests.

^e^ only 10 patients in the non-NPV exercise group completed the SGRQ at 12 weeks.

The improvement in 6MWD in the NPV-exercise group was accompanied by an improvement in resting dyspnea sensation as determined by the modified Borg scale (Table 1, 1.4 ± 1.5 points vs. 0.8 ± 1.3 points, *P* = 0.049). In contrast, there was no change in Borg scale (1.3 ± 0.4 points vs. 1.6 ± 0.4 points, *P* = 0.573) in the control group. Both groups of patients suffered from O_2_ desaturation during 6MWT, which was not improved in either group at 12 weeks.

### Changes in pulmonary function

**Figure 2:**
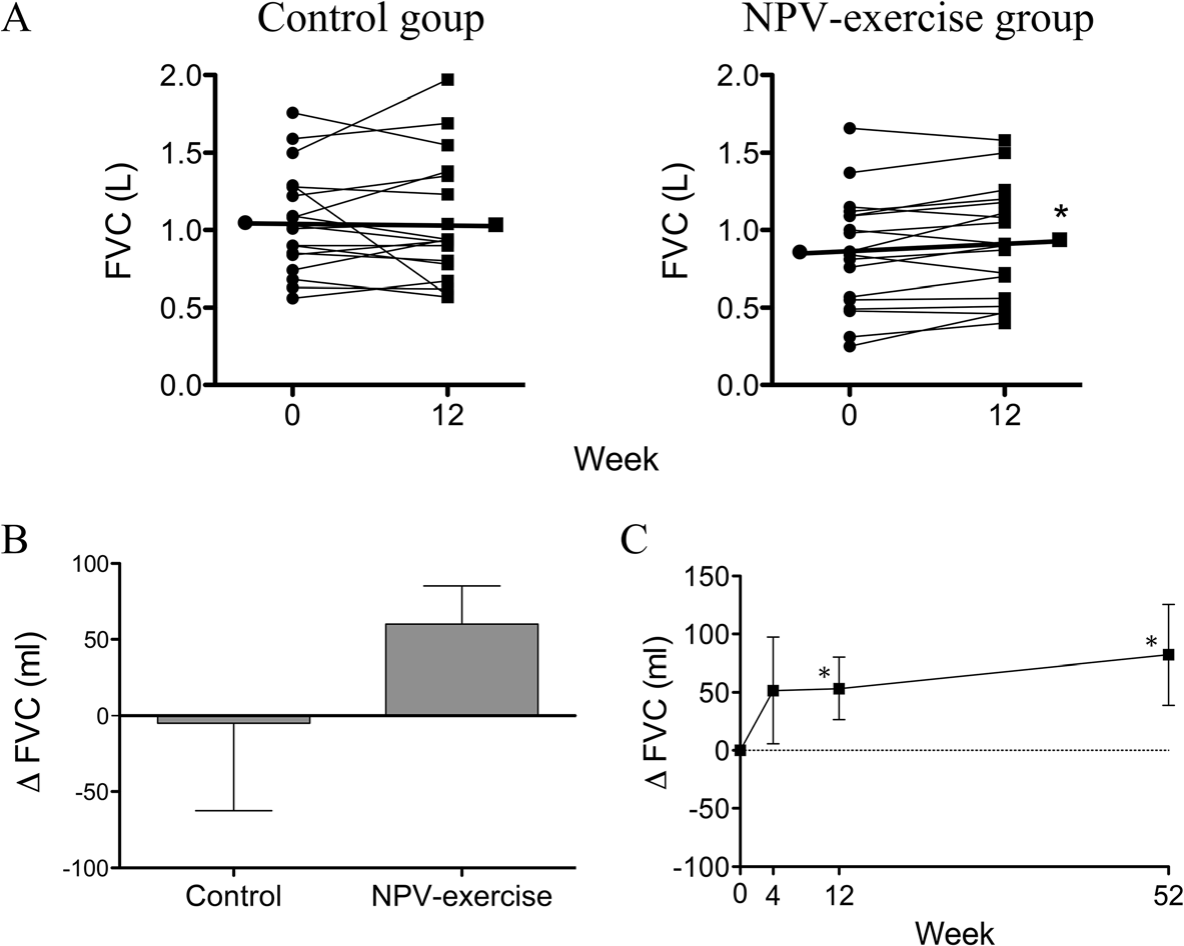
**Effects of NPV-exercise training vs. control on FVC. A.** Individual changes of FVC in both groups before and after the 12-week course (Control, *n* = 18; NPV-exercise, *n* = 18). **B.** Comparisons of delta-FVC (ΔFVC; changes from the base line after 12 weeks; mean ± SD) between both groups (Control, *n* = 18; NPV-exercise, *n* = 18). **C.** Time course of FVC in the NPV-exercise group (*n* = 14) during the 12 months.

**Figure 3:**
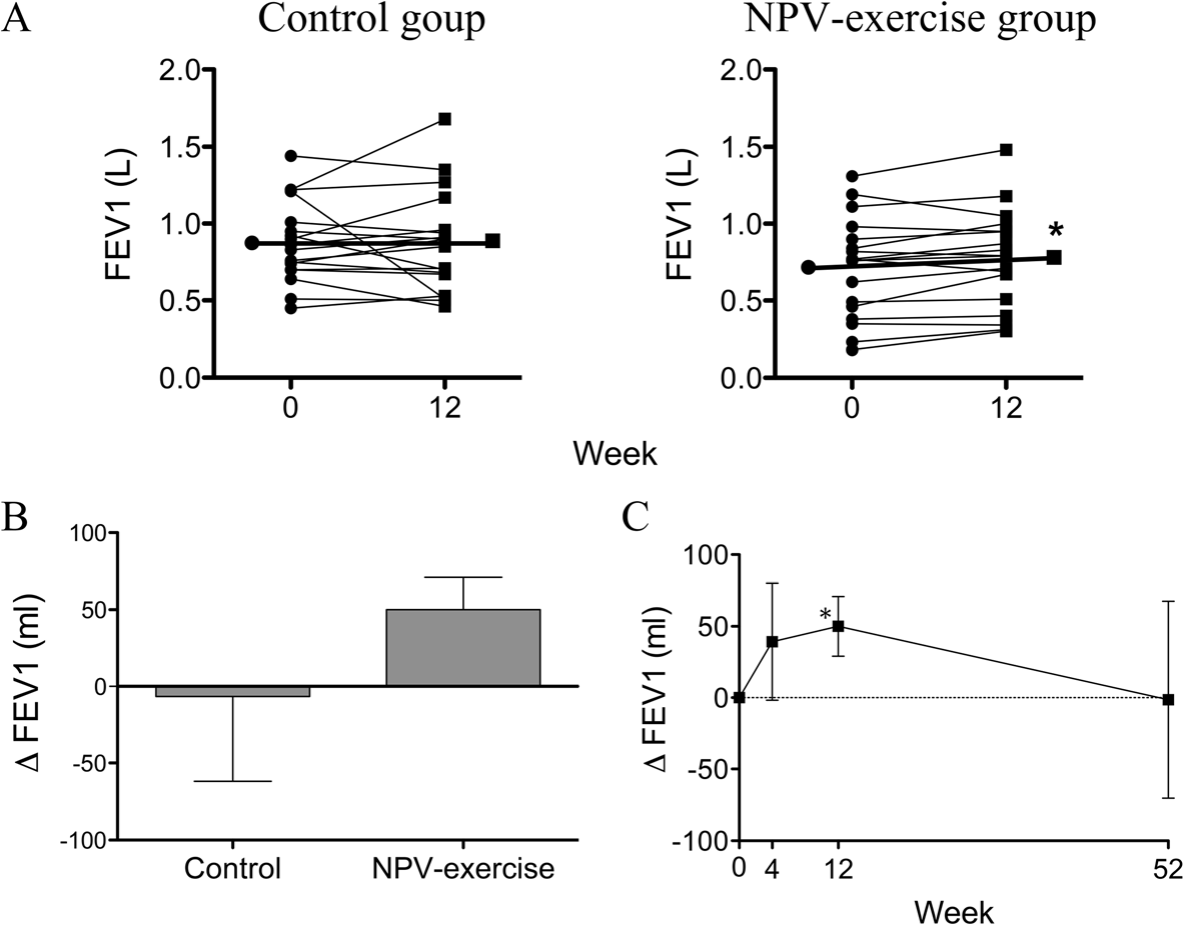
**Effects of NPV-exercise training vs. control on FEV**_**1**_**. A.** Individual changes of FEV_1_ in both groups before and after the 12-week course (Control, *n* = 18; NPV-exercise, *n* = 18). **B.** Comparisons of delta-FEV_1_ (ΔFEV_1_; changes from the base line after 12 weeks; mean ± SD) between both groups (Control, *n* = 18; NPV-exercise, *n* = 18). **C.** Time course of ΔFEV_1_ in the NPV-exercise group (*n* = 14) during the 12 months.

Pulmonary function was improved in the NPV-exercise group after 12 weeks of training (Table 2, Figures 2A and 3A); FVC improved by 53.3 ± 26.8 ml (0.85 ± 0.09 L vs. 0.91 ± 0.08 L, *P* = 0.029), and FEV_1_ improved by 50.0 ± 21.0 ml (0.72 ± 0.08 L vs. 0.77 ± 0.08 L, *P* = 0.029), being equivalent to increases of 2.5 ± 1.0% predicted and 3.4 ± 1.2% predicted, respectively. In contrast, pulmonary function did not change in the control group (Table 2 and Figures 2A and 3A). However, the between group differences in either ΔFVC or ΔFEV_1_ did not reach statistical significance (Figures 2B and 3B). Both pulmonary functions in the NPV-exercise group had a trend of increase at 4 weeks, which became significant at the end of 12-week training program (Figures 2C and 3C). Whilst FEV_1_ declined to a level similar to that at baseline at 52 weeks (Figure 3C), the increase in FVC was sustained at this time point (Figure 2C). The ratio of FEV_1_/FVC did not change in either group at 12 weeks compared with that at baseline (Table 2).

### Changes in health-related quality of life

**Figure 4:**
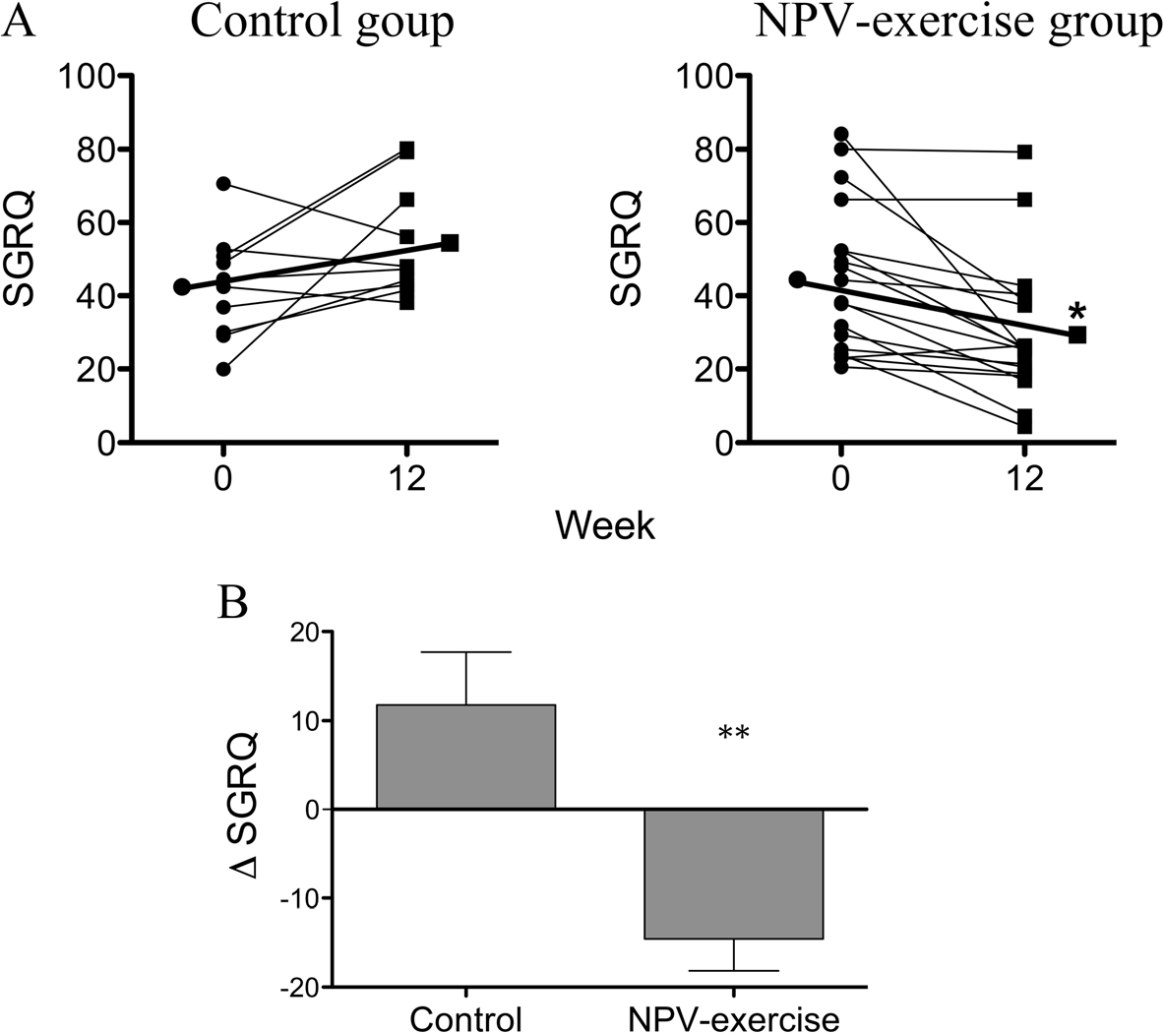
**Effects of NPV-exercise training vs. control on health-related quality of life. A.** Individual changes of SGRQ total scores in both groups before and after the 12-week course (Control, *n* = 10; NPV-exercise, *n* = 18). **B.** Comparisons of St. George’s Respiratory Questionnaire (SGRQ) total score (ΔSGRQ; changes from the base line after 12 weeks mean ± SD) between both groups (Control, *n* = 10; NPV-exercise, *n* = 18).

All the 18 patients in the NPV-exercise group completed the SGRQ test, whereas only 10 out of the 18 patients in the control group did. The other 8 patients did not receive the test for personal reasons. The total SGRQ score at 12 weeks decreased by 14.5 ± 3.6 (Table 2 and Figure 4A, 44.6 ± 20.3 vs. 30.0 ± 18.8, *P* = 0.009) compared with that at baseline in the NPV-exercise group. Improvements in SGRQ were consistently seen in the symptom score (54.6 ± 21.0 vs. 24.5 ± 16.8, *P* < 0.001), the activity score (53.7 ± 24.6 vs. 45.9 ± 23.7, *P* = 0.048), and the impact score (36.2 ± 24.2 vs. 22.6 ± 21.6, *P* = 0.077). Ten patients in the control group completed the SGRQ. An improvement was not seen in this group, and the impact score even deteriorated (30.0 ± 13.3 vs. 46.7 ± 22.2, *P* = 0.04). The between group difference in ΔSGRQ was significant (Figure 4B, *P* < 0.01).

### Effects of NPV-exercise training vs. Control in patients with interstitial lung disease and patients with chest wall deformities

To see whether NPV-exercise training benefited distinct subgroups of patients, results were separately analyzed for patients with interstitial lung disease (ILD) and chest wall deformities (CWD) (Table 3). FVC and FEV_1_ increased by 4.2 ± 4.1% predicted (36.3 ± 13.0% vs. 40.5 ± 11.6%, *P* < 0.01) and 5.1 ± 5.5% predicted 40.9 ± 15.8% vs. 45.9 ± 15.2%, *P* < 0.05) in patients with ILD in the NPV-exercise group (*n* = 10). The between-group difference was significant in FVC (*P* < 0.01). These increases were not seen in CDW patients in the NPV-exercise group (*n* = 8). 6MWD increased by 45.8 ± 87.5 m (352.3 ± 52.8 m vs. 399.5 ± 45.1 m, *P* < 0.05) in ILD patients in the NPV-exercise group (*n* = 10). The between-group difference was significant (*P* < 0.05). A trend of increase was also seen in CDW patients (*n* = 8, by 17.8 ± 52.2 m, 395.0 ± 99.9 m vs. 412.8 ± 109.8 m). By contrast, both subgroups of patients had a trend of disease in 6MWD by 29.7 ± 74.3 m (*n* = 12) and 47.2 ± 54.2 m (*n* = 6), respectively, in the control group. NPV-exercise training improved the total scores of SGRQ in patients with ILD (*n* = 10) and CWD (*n* =8) by 17.5 ± 18.5 (54.5 ± 21.3 vs. 37.0 ± 21.1, *P* < 0.05) and 10.9 ± 10.2 (32.2 ± 10.0 vs. 21.3 ± 11, *P* < 0.05). This score had a trend of deterioration in both subgroups by 1.03 ± 20.1 (*n* = 8) and 17.8 ± 16.6 (*n* = 2) in the control group. The between-group difference was significant for ILD (*P* < 0.01) and CWD (*P* < 0.05).

**Table 3:** Changes in pulmonary function, 6-minute walk test, and health-related quality of life before and after 12 weeks of pulmonary rehabilitation in patients with interstitial lung disease vs. chest Wall disease

	**Non-NPV-exercise group**		**NPV-exercise group**	
	**Baseline**	**12 weeks**	**Baseline**	**12 weeks**
FVC, % predicted				
ILD	40.3 ± 10.3	38.4 ± 13.5	36.3 ± 13.0	40.5 ± 11.6^b, c^
Chest wall	32.7 ± 8.6	37.0 ± 14.5	28.0 ± 8.5	28.3 ± 7.4
FEV_1_, % predicted				
ILD	45.6 ± 11.8	43.8 ± 18.2	40.9 ± 15.8	45.9 ± 15.2^a^
Chest wall	35.3 ±11.3	44.2 ± 20.2	31.9 ± 8.6	33.3 ± 8.0
FEV_1_/FVC, %				
ILD	81.8 ± 6.8	84.4 ±7.9	82.3 ± 9.7	81.0 ±11.8
Chest wall	88.8 ± 10.6	82.2 ± 8.1	83.8 ± 9.7	86.6 ± 9.1
6MWD, m				
ILD	385.2 ± 82.6	355.3 ± 85.0	352.3 ± 52.8	399.5 ± 45.1^a^
Chest wall	318.2 ± 122.4	288.0 ± 99.3	395.0 ± 99.9	412.8 ± 109.8
SaO2, % pre/post 6MWT				
ILD	94.3/ 78.7	93.1/ 78.6	96.0/80.5	96.3/83.1
Chest wall	94.8/ 77.3	93.3/ 73.3	91.3/72.6	92.4/74.1
Borg, pre/ post 6MWT				
ILD	0.9/ 5.1	1.5/5.8	1.7/ 5.5	0.8/4.8
Chest wall	2.0/ 5.8	1.7/5.8	1.1/ 5.3	0.8/5.0
SGRQ				
Symptom score				
ILD	47.6 ± 21.5^c^	42.1 ± 19.3^c^	61.7 ± 16.6^e^	29.5 ± 19^b,e^
Chest wall	50.3 ± 5.8^d^	64.1 ± 0.2^d^	53.7 ± 5.8^f^	45.9 ± 5.6^a,f^
Active score				
ILD	62.2 ± 21.5	73.7 ± 12.8	61.4 ± 27.4	53.5 ± 23.2
Chest wall	59.5 ± 0.0	66.4 ± 18.8	43.9 ± 17.6	36.5 ± 22.0
Impacts score				
ILD	29.3 ± 13.6	43.9 ± 19.2	48.3 ± 25.3	30.0 ± 25.3^a^
Chest wall	32.9 ± 16.7	58.0 ± 38.9	21.2 ± 11.7	13.5 ± 12.2
Total score				
ILD	42.3 ± 16.0	52.6 ± 14.0	54.5 ± 21.3	37.0 ± 21.1^a^
Chest wall	43.8 ± 9.8	61.6 ± 26.4	32.2 ± 10.0	21.3 ± 11.5^a^

Values are mean ± SD.

^a^ p < 0.05, baseline vs. 12 weeks; ^b^ p < 0.01, baseline vs. 12 weeks; ^c^*n* = 8; ^d^*n* = 2; ^e^*n* = 10; ^f^*n* = 8.

## Discussion

The present study investigated the effect of exercise training under the support of NPV for patients with severe RLD. This study was designed in a prospective controlled setting. The between group difference in Δ6MWD, the primary end point, reached statistical difference in favor of the NPV-exercise group. An improvement in SGRQ was also seen in the NPV-exercise group compared with the control group.

Although exercise training PR programs can improve exercise capacity and HRQoL in patients with RLD for those with severe disease [8], it tends to be intolerable. The patients enrolled for PR training in the present study (FVC 32.5 ± 11.7% predicted) appeared to be more severe than those in previous reports (FVC 48–68% predicted) [7-9]. Almost none of the patients were able to perform functional exercise tasks because of severe ventilation impairment accompanied with muscle weakness and deconditioning. Muscle weakness can be overcome by ventilation support as demonstrated by Borel et al [12]. With the assistance of NPV, all patients in the study group well tolerated the exercise PR program, and they also achieved even better results (difference in 6MWT between groups 66.6 m) than those demonstrated in earlier reports (35.0–46.3 m) [7,8]. This difference is greater than the minimal clinical important difference (MCID) for COPD (54 m) [21] and that for idiopathic pulmonary fibrosis (24–45 m) [22]. Nevertheless, our patients comprised a heterogeneous group of patients with different diseases, for most of which the MCID has not been defined. Whether the improvement in exercise capacity reaches clinical significance needs further study to confirm.

Ventilation support by using NPPV can unload ventilator muscles, leading to a reduction in breathing work and dyspnea sensation [23]. This unloading has also been demonstrated to improve peripheral muscle oxygenation in the absence of changes in systemic oxygen delivery in patients with advanced COPD during high-intensity exercise, probably due to redistribution of cardiac output to appendicular muscles [24]. This will not only allow a greater intensity of exercise but also allow it to be sustained for longer periods to achieve a training effect [11]. Although not proven directly, NPV may also benefit patients with severe RLD through similar mechanisms such that patients may be able to tolerate exercise training to an extent of adequate intensity. Increased peripheral muscle oxygenation may also contribute to a better training efficacy. Although more data is necessary to support this theory, this concept may be a rationale to introduce NPV during exercise training for patients with RLD with less severity, or even other respiratory diseases such as COPD.

The present study showed that an NPV-assisted exercise program remarkably improved health-related quality of life (HRQoL), as demonstrated by the SGRQ scores. Patients in the NPV-exercise group had clinically significant decreases in total and almost all components of the scores, e.g. symptoms, activity and impact. This is consistent with most previous reports observing the effects of exercise training on HRQoL in a range of RLD using the Chronic Respiratory Disease Questionnaire [8,9] or the SGRQ [7]. Both resting and post-exercise dyspnea sensation, determined by the Borg score, also improved. This improvement was not associated with changes of muscle power or O_2_ saturation. Interestingly, we observed a significant increase in pulmonary function, including FEV_1_ and FVC, after 12 weeks of training. Importantly, the increase in FVC (2.5% predicted) reached the recently defined minimal clinical important difference for IPF [25], in which the small changes of 2–6% were thought to be clinically important. This was not seen in previous reports on exercise training for RLD patients [8,9]. Further study is needed to see whether this improvement in pulmonary function has clinical significance. Of note, Smith et al. reported their experience of treating kyphotic patients with nocturnal non-invasive ventilation, either with an individually constructed cuirass shell and a negative pressure pump or nasal intermittent positive pressure ventilation, long-term use of which resulted in a significant increase in FEV_1_ and FVC [26]. As the between group difference in FVC in the present study, albeit with a trend, was not statistically significant, further studies enrolling more patients is necessary to confirm our observations.

The present study did not include a group of NPV without endurance training. It is therefore not conclusive whether the study group got benefits from NPV and/or exercise training. Guzun *et al.* recently reported that physical training at mild intensity, even below the level expected for a physiological training effect, can induce comparable changes in skeletal muscles oxidative energy metabolism in patients with COPD and sedentary healthy subjects [27]. Thus it is interesting to conduct a study to explore the pure effect of NPV or NPV with physical activity of mild intensity.

A limitation of the study is the non-randomized design. This is dictated by the fact that few patients were willing to be randomized in such kind of intervention. Nevertheless, the baseline characteristics were similar between the two groups such that a skewed population in each group, although cannot be completely excluded, became less likely. In addition, there is heterogeneity of patients. Subgroup analysis revealed that NPV-exercise training benefited both ILD and CWD patients in HRQoL. Whereas this training improved exercise capacity and pulmonary functions in ILD patients, it only had a trend of improvement (exercise capacity) or no effects (pulmonary functions) in CWD patients. Whether this training has clinically relevant effects on exercise capacity in these patients needs to be confirmed in a larger scale of study.

## Conclusions

Ventilation support with NPV during exercise training is feasible for patients with severe RLD who are profoundly intolerant to exercise. In such patients, exercise training with NPV support increases exercise capacity and HRQoL to a level of clinical importance, at least in patients with ILD. Thus, NPV should be considered for patients with severe RLD who are not able to tolerate exercise training with adequate intensity. Our study also raises the possibility that NPV enhances the training effect of exercise through redistribution of cardiac output to the limb muscles, and that NPV increases pulmonary functions. Further studies to confirm these findings are warranted and may potentially extend the use of NPV in pulmonary rehabilitation for patients with RLD, and potentially for other chronic conditions with impairment in exercise tolerance.

## Competing interests

The authors have no conflicts of interest to disclose.

## Authors’ contribution

SCH conceived the idea, designed, carried out the study, performed the statistical analysis and were the main writers of the manuscript. HCL and HPK directed and supervised the study. LFC, TFS, WCJ and CHW assisted in subjects recruiting and helped to carry out the study. KYL directed the statistical analysis, data interpretation and manuscript preparation and writing. All authors read and approved the final manuscript.

## Acknowledgments

The authors wish to thank the patients and personnel of the hospital unit for their cooperation during the course of this study.

## Funding

This work was supported by grants to S.-C. Ho. from Chang Gung Memorial Hospital (CMRPG371891).
